# Clinical Characteristics of Pediatric Cases of COVID-19 in Hunan, China: A Retrospective, Multi-Center Case Series

**DOI:** 10.3389/fped.2021.665377

**Published:** 2021-09-24

**Authors:** Lei Wu, Xiao-Fo Zhang, Yong Yang, Xiu-Ying Yi, Xin-Ping Jiang, Hai-Ying Han, Xiao-Ying Cao, Tuan-Mei Wang, Man-Zhi Wang

**Affiliations:** ^1^Department of Pediatrics, Changsha Central Hospital, University of South China, Changsha, China; ^2^Department of Critical Care Medicine, Changsha Central Hospital, University of South China, Changsha, China; ^3^Department of Pediatrics, Zhuzhou Central Hospital, Zhuzhou, China; ^4^Department of Pediatrics, Loudi Central Hospital, Loudi, China; ^5^Department of Pediatrics, Shaoyang Central Hospital, Shaoyang, China; ^6^Department of Infectious Disease, Chenzhou Second People's Hospital, Chenzhou, China; ^7^Department of Pediatrics, Changsha Hospital for Maternal and Child Health Care of Hunan Normal University, Changsha, China

**Keywords:** COVID-19, pediatric infection, epidemiology, symptoms, laboratory characteristics

## Abstract

**Objective:** Aimed to investigate the epidemiological characteristics, clinical features, treatment, and short-term prognosis of COVID-19 in children.

**Methods:** Retrospective analysis was conducted in 48 children with COVID-19 admitted to 12 hospitals in eight cities in Hunan province, China, from January 26, 2020 to June 30, 2020.

**Results:** Of the 48 cases, Familial clusters were confirmed for 46 children (96%). 16 (33%) were imported from other provinces. There were 11 (23%) asymptomatic cases. only 2 cases (4%) were severe. The most common symptom was fever (*n* = 20, 42%). Other symptoms included cough (*n* = 19, 40%), fatigue (*n* = 8, 17%), and diarrhea (*n* = 5, 10%). In the early stage, the total peripheral blood leukocytes count increased in 3(6%) cases and the lymphocytes count decreased in 5 (10%) cases. C-reactive protein and procalcitonin were elevated respectively in 3 (6%) cases and 2 (4%) cases. There were abnormal chest CT changes in 22 (46%) children, including 15 (68%) with patchy ground glass opacity, 5 (22%) with consolidation, and 2 (10%) with mixed shadowing. In addition to supportive treatment, antiviral therapy was received by 41 (85%) children, 11 (23%) patients were treated with antibiotics, and 2 (4%) were treated with methylprednisolone and intravenous immunoglobulin. Compared to 2 weeks follow-up, one child developed low fever and headache during the 4 weeks follow-up, 3 (6%) children had runny noses, one of them got mild cough, and 4 (12%) children had elevated white blood cells and lymphocytes. However, LDH and CK increased at 2 weeks and 4 weeks follow-up. 2 weeks follow-up identified normal chest radiographs in 33 (69%) pediatric patients. RT-PCR detection of SARS-CoV-2 was negative in all follow-up patients at 2 and 4 weeks follow-up. All 48 pediatric patients were visited by calling after 1 year of discharge.

**Conclusions:** Most cases of COVID-19 in children in Hunan province were asymptomatic, mild, or moderate. Close family contact was the main route of infection. It appeared that the younger the patient, the less obvious their symptoms. Epidemiological history, nucleic acid test, and chest imaging were important tools for diagnosis in children.

## Introduction

The emergence and spread of a novel coronavirus (SARS-CoV-2) in Wuhan, Hubei province, China in December 2019 has resulted in a global health crisis, threatening many thousands of lives. The disease caused by SARS-CoV-2 was officially called coronavirus disease-2019 (COVID-2019), and the World Health Organization (WHO) has declared it to be a pandemic. It has high contagiousness and rapid spread through human-to-human transmission (1). As of June30, 2020, COVID-19 had been reported in more than 200 countries, affecting over 10 million individuals and resulting in more than 500,000 deaths (2). Clusters are an important epidemiological characteristic of this outbreak (3). At the emerging stage, COVID-19 infection starts with person-to-person transmission within a community, almost exclusively in adults. Intrafamilial transmission causes further spread of the virus within families, especially to the elderly and children, who are vulnerable to the infection (4). Although current reports indicate that most pediatric cases are mild compared with cases in adults (5), the risk of lethality still exists. During previous SARS and MERS outbreaks, acute respiratory distress syndrome (ARDS) and deaths occurred in children (6–8).

By June30, 2020, a total of 83,534 laboratory-confirmed cases had been documented in China (9). However, relatively few pediatric COVID-19 cases have been confirmed. In a review of 44,672 laboratory-confirmed cases from across China as of February 11, 2020, only 416 (0.9%) occurred in patients below 10 years of age and only 549 (1.2%) in patients between 10 and 20 years of age (4). Moreover, a study with 72,314 cases reported that less than 1% cases were in children under the age of 10 years (10). Although extensive research has been undertaken on COVID-2019 since the outbreak, little information is available regarding the epidemiological characteristics and clinical features in children.

Hunan province borders on Hubei province, the capital of which, Wuhan, was the epicenter of COVID-19 in China. Cases in Hunan province may therefore provide evidence of COVID-19 clinical course on children. In order to provide guidance for clinical prevention and treatment, this study retrospectively analyzed the epidemiological history and clinical manifestations of COVID-19 in children, in addition to the treatment and prognosis of children infected, in eight different cities of Hunan province.

## Methods

### Data Sources

This retrospective review was conducted in 12 participating hospitals (The First Hospital of Changsha; The First People's Hospital of Changsha County; Zhuzhou Central Hospital; Zhuzhou First People's Hospital; Loudi Central Hospital; Shaoyang Central Hospital; Shaoyang First People's Hospital; Chenzhou Second People's Hospital; Xiangtan Central Hospital; The First Hospital of YueYang; Changde Second People's Hospital; Nanhua Hospital, University of South China) from eight cities in Hunan province of China. We included all children under 18 years of age who were diagnosed with COVID-19 between January 26 and June30, 2020. Cases of COVID-19 were laboratory-confirmed by a real-time reverse transcriptase-polymerase chain reaction (RT-PCR) assay of nasal and pharyngeal swab specimens in the laboratory of the Chinese CDC.

### Clinical Assessment and Disease Severity Grouping

We followed the recommendations issued by the Pediatric Society of the Chinese Medical Association for the diagnosis, prevention, and control of COVID-19 in children (first interim edition) (11). We collected data on age, gender, exposure history, comorbidities, clinical symptoms, laboratory results, chest radiological features, and treatments. Patients were divided into five groups: (1) Asymptomatic: no symptoms and no pneumonia on imaging; (2) Mild: only mild clinical symptoms including upper respiratory symptoms and some slight gastrointestinal symptoms with no pneumonia on imaging; (3) Moderate (referred to as “ordinary/common” in the aforementioned pediatric society's recommendations): common symptoms and signs with typical pneumonia on imaging, or no clinical symptoms and signs but pneumonia on imaging; (4) Severe: the above typical features accompanied by dyspnea and hypoxemia (oxygen saturation ≤ 92%); and (5) Critical: patients rapidly progressing to ARDS or respiratory failure, with shock, multiple organ dysfunction (MODS), and/or death.

### Assessment at Follow-Up

Pediatric patients were followed up at 2 weeks, 4 weeks and 1yearrespectively after discharge. At follow-up clinical manifestations, laboratory tests, RT-PCR detection of SARS-CoV-2 and CT scans were implemented at 2 weeks and 4 weeks after discharge. Phone calls were completed at 1 year after discharge. All follow-up procedures were performed according to Chinese clinical protocol for COVID-19 (Edition 8) (12).

### Statistical Analysis

Data were processed by SPSS 19.0 statistical software. Differences between groups were compared using Chi-square tests and Fisher's exact probability method. Normal distribution measurement data are described by mean and standard deviation ($\bar{x}\pm s$). *P* < 0.05 was considered statistically significant.

## Results

### Epidemiological Characteristics

**Figure 1:**
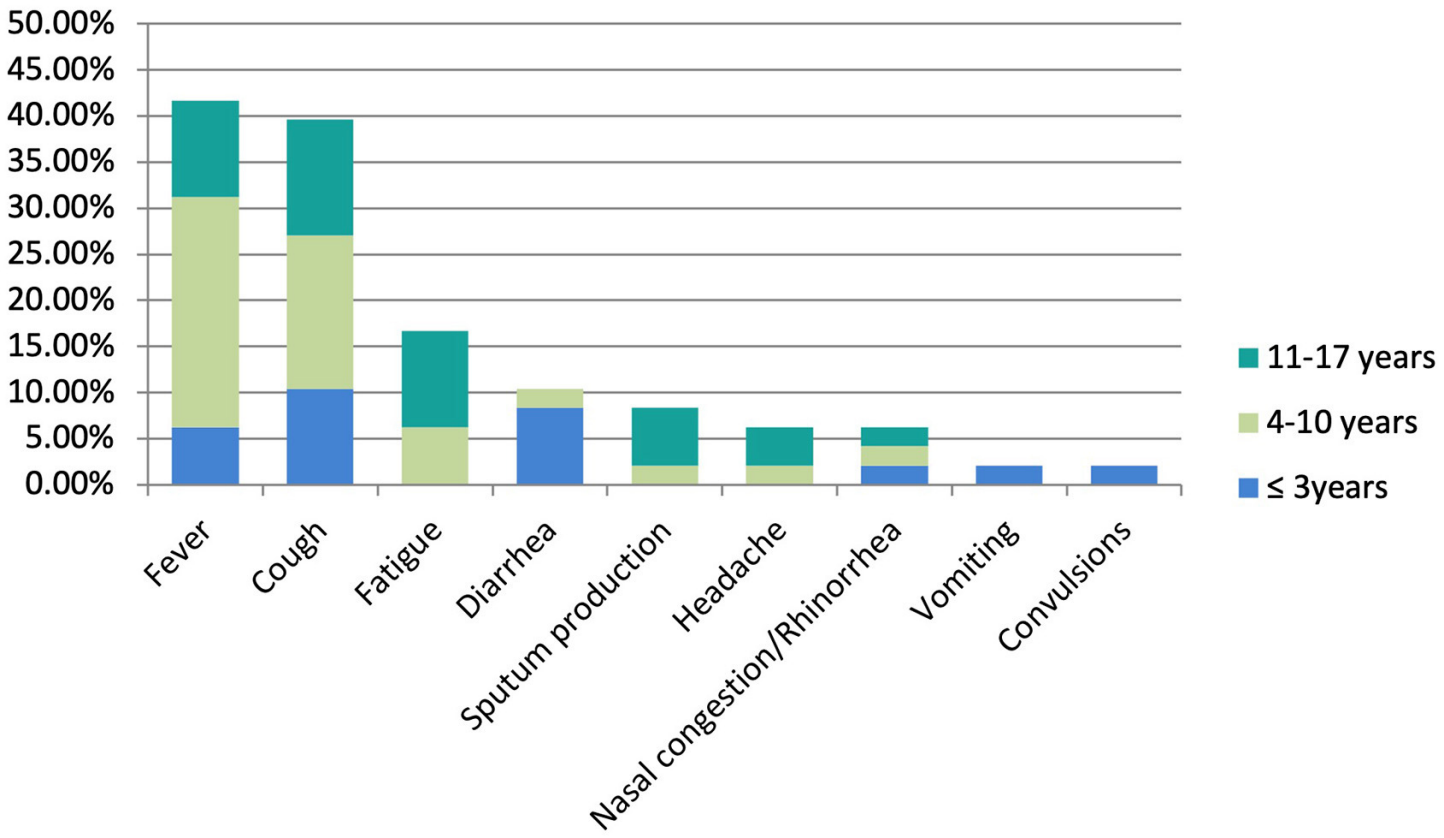
Bar plot of percentage of the symptom presentation in different age groups.

A total of 48 pediatric cases of COVID-19 from eight cities (Changsha, Zhuzhou, Loudi, Shaoyang, Hengyang, Chenzhou, Changde, and Yueyang) in Hunan province were enrolled in this study. The distribution of age and sex as well as disease severity are shown in Table 1. The mean age of the 48 pediatric patients was 8.27 ± 4.66 years, ranging from 7 months to 17 years. Among these patients, only 2 (4%) wereunder 1 year of age; 11 (23%) were 1 to 3 years of age; 18 (38%) were 4 to 10 years of age; and 17 (35%) were 11 to 17 years of age. Males comprised 52% of the patients, and therefore the ratio of male to female patients was almost 1:1. None of the patients had underlying diseases, and all of them had documented history of exposure to a positive case. Imported cases comprised 33% (16 children). Seven of these children had lived in Wuhan for a long time, Eight of then were from cities in Hubei province other than Wuhan, and one child was infected on a cruise ship from the port of Guangzhou, China between January 19 and 24, 2020. There was no history of exposure to the Wuhan Huanan Seafood Wholesale Market for any child. Familial clusters were confirmed for 46 children (96%). Only two children (4%) had not been exposed to any confirmed adult patients; however, each of these children was imported case and had lived in Wuhan for a long time. Percentage of the symptom presentation in different age groups is shown in Figure 1.

**Table 1:** Demographic characteristics and disease severity distribution of 48 children infected with COVID-19.

	**Asymptomatic**	**Mild**	**Moderate**	**Severe**	**Total (%)**	**χ^2^**	***P***
**Sex (%)**							
Male	5 (10)	9 (19)	10 (21)	1 (2)	25 (52)	0.907#	0.900
Female	6 (13)	6 (13)	10 (20)	1 (2)	23 (48)		
**Age group (%)**							
< 1 year	1 (2)	0	1 (2)	0	2 (4)	11.779#	0.152
1–3 years	4 (8)	1 (2)	5 (10)	1 (2)	11 (23)		
4–10 years	1 (2)	9 (19)	7 (15)	1 (2)	18 (38)		
11–17 years	5 (10)	5 (10)	7 (15)	0	17 (35)		
Total	11 (23)	15 (31)	20 (42)	2 (4)	48 (100)		
**Epidemiological**							
Contactwith local case	10 (21)	13 (27)	9 (19)	0	32 (67)		
Imported from Hubei (except Wuhan)	1 (2)	1 (2)	5 (10)	1 (2)	8 (16)		
Contact with Wuhan personnel	0	2 (4)	4 (8)	1 (2)	7 (15)		
Vechile (airpalne, coach,train, ship)	0	0	1 (2)	0	1 (2)		
**Family contact**							
Local cases	10 (21)	13 (27)	9 (19)	0	32 (67)		
Imported cases	1 (2)	1 (2)	11 (23)	1 (2)	14 (29)		

*#used Fisher exact probability method*.

### Clinical Features

As shown in Table 1, 11 children (23%) were diagnosed with asymptomatic infection, 15 cases (31%) were diagnosed as mild, and 20 cases were diagnosed as moderate (42%), including 3 subclinical cases. Only two (4%) patients had severe disease, and no cases were critical. As shown in Table 2, the most common symptom was fever, occurring in 20 cases (42%). Of these, 13 children had low-grade fever (37.3–38°C), and 3 had high fever (>39°C). In 28 children (58%), normal body temperature was maintained throughout the course of COVID-19. Eight children (17%) experienced fatigue, three (6%) had headaches, and three (6%) had nasal congestion/runny noses. Coughing was another common symptom, present in 40% of cases, including four children (8%) who had coughing with sputum. Six children (13%) experienced gastrointestinal symptoms, including diarrhea (five cases) and vomiting (one case), and one child presented with diarrhea as the only symptom in the course of the disease. One patient with high fever (>39°C) developed convulsions, which did not recur after the fever had gone. No children had discomfort such as dyspnea, hemoptysis, or altered mental status, and none required intensive care support or invasive mechanical ventilation. Accurate and detailed information on the clinical signs of the children is not available due to the difficulty for medical staff wearing protective clothing of carrying out detailed physical examinations, especially for the lung auscultation.

**Table 2:** Clinical features and radiographic findings of 48 children with COVID-19 and follow-up examination of 33 pediatric patients after 2 weeks and 4 weeks discharge.

	**≤3 years (*n =* 13)**	**4–10 years(*n =* 18)**	**11–17 years (*n =* 17)**	**Total (%)**	**2 weeks follow-up (*n =* 33)**	**4 weeks follow-up(*n =* 33)**	**χ^2^**	***P***
**Symptoms**								
**Fever**	3	12	5	20 (42)	0	1	7.527	0.023^*^
37.3-38°C	1	5	7	13 (27)			5.675#	0.174
38.1-39°C	1	2	1	4 (8)				
>39°C	2	1	0	3 (6)				
Fatigue	0	3	5	8 (17)	0	0	4.483#	0.101
Headache	0	1	2	3 (6)	0	1	1.492#	0.620
Nasal congestion/Rhinorrhea	1	1	1	3 (6)	0	3	0.521#	1.000
Cough	5	8	6	19 (40)	0	1	0.315	0.931
Sputum production	0	1	3	4 (8)	0	0	2.583#	0.362
Vomiting	1	0	0	1 (2)	0	0	2.304#	0.271
Diarrhea	4	1	0	5 (10)	0	0	6.432#	0.024^*^
Convulsions	1	0	0	1 (2)	0	0	2.304#	0.271
**Chest CTscan**					NA	NA		
Ground glass opacity	5	5	5	15 (68)			2.594#	0.710
Consolidation	1	3	1	5 (23)				
Mixed shadowing	1	0	1	2 (11)				
Lesions on one lobe	2	7	3	12 (55)			5.634	0.061
Lesions on two and more lobes	5	1	4	10 (45)				
**chest radiographs(*****n*** **=** **33)**					0	NA		
**Laboratory tests**								
**White blood cell count**								
4.0-10.0 × 10^9^ /L	10	18	15	43 (90)	32	28		
< 4.0 × 10^9^ /L	0	0	2	2 (4)	0	0		
>10.0 × 10^9^ /L	3	0	0	3 (6)	1	4		
**Lymphocyte count**								
< 1.5 × 10^9^ /L	0	3	2	5 (10)	0	0		
>3.2 × 10^9^ /L	6	2	2	10 (21)	1	4		
PLT < 100 × 10^9^/L	0	0	0	0	0	0		
Albumin < 40 g/L	0	0	3	3 (6)	0	0		
ALT>40 U/L	0	0	0	0	0	0		
AST >40 U/L	0	0	0	0	0	0		
Cr>73 μmol/L	0	0	0	0	0	0		
Urea nitrogen >7.14 mmol/L	0	0	0	0	0	0		
CK≥200 U/L	0	0	0	0	1	5		
D-dimer≥0.5 mg/L	2	0	0	2 (4)				
LDH≥250 U/L	1	0	2	3 (6)	3	4		
BS> 7.0 mmol/L	0	0	0	0	0	0		
CRP≥10 mg/L	1	2	0	3 (6)	0	2		
PCT≥0.5 ng/ml	1	1	0	2 (4)				
Serum iron protein >400 ng/ml	0	0	0	0				

*#used Fisher exact probability method*,

* *P < 0.05. PLT, Blood platelet; ALT, Alanine aminotransferase; AST, Aspartate aminotransferase; Cr, Serum creatinine; CK, Creatine kinase; LDH, Lactate dehydrogenase; BS, Blood glucose; CRP, C-reactive protein; PCT, Procalcitonin*.

### Laboratory Findings

All patients underwent routine peripheral blood counts and coagulation, myocardial enzyme, and liver and kidney function tests. Compared to adults, children rarely have low levels of leucocyte and lymphocytes. The normal range of leucocytes was present in 43 patients (90%), 3 patients (6%) had elevated levels, and 2 (4%) had low levels. Lymphopenia (lymphocyte count < 1.5 × 10^9^ per liter) was present in 5 patients (10%), while 43 (90%) had normal lymphocyte counts. All patients in this study had normal platelets and hemoglobulin. Three children (6%) were found to have elevated C-reactive protein (CRP) (>10 mg/l). Elevated levels of procalcitonin (>0.5 ng/ml) and D-dimer (≥0.5 mg/L) were detected in two severecases (4%). All children in this study had normal levels of alanine aminotransferase, aspartate aminotransferase, creatine kinase, serum creatinine, urea nitrogen, and blood glucose. Serum ferritin and immune globulin were measured in 11 and 7 children, respectively; all levels were normal.

### Radiographic Findings

Of the 48 patients, 22 (46%) showed lesions on chest CT scan (Table 2). Among these, 12 patients (55%) had one-lobe involvement, and in 10 (45%) two or more lobes were involved. The most common radiographic finding was ground glass opacity, which was present in 15 patients (68%); 5 patients (22%) had consolidation, and 2 patients (10%) had mixed shadowing of ground glass and consolidation. None of the patients had pleural effusion. For 20 patients, chest CT examination was repeated after three to seven days. Of these 20, the second scan revealed no changes in 4 patients (20%), disease resolution in 11 (55%), and disease progression in 5 (25%). All the imaging lesions of the 20 patients disappeared after two weeks of medical treatment.

### Medical Treatments

Of the 48 children in this study, 41(85%) received antiviral therapy. Among these, 24 children (59%) were treated with a single antiviral drug. Interferon was given to 9 children (atomized or sprayed into the nasal cavity, for 7 to 10 days); lopinavir/ritonavir (LPV/RTV) was given to 12 children; two children received Arbidol (oral, three times a day for 7 to 14 days), and one child received ribavirin (oral, twice a day for 10 days). The remaining children were treated with combinations of two or more antiviral drugs, including oseltamivir phosphate (oral, twice a day for 5 days), interferon, LPV/RTV, Arbidol, ribavirin, and traditional Chinese medicine. Eleven children (23%) were treated with antibiotics, including six who received azithromycin (oral, 10 mg/kg per day for 3 to 5 days) for mycoplasma infection. Two children (4%) were treated with methylprednisolone (intravenous, 2 mg/kg per day for 3 days) and intravenous immunoglobulin (IVIG) (intravenous, 1 g/kg per day for 2 days) for persistent high fever and pulmonary disease progression. None of the children received mechanical ventilation.

### Follow-Up Examination

Of the 48 discharged patients, 33 (69%) patients were successfully followed for collection of symptom details and laboratory results after 2 weeks and 4 weeks since discharge. Within 2 weeks after discharge, clinical symptoms were resolved in all follow-up patients. White blood cells and lymphocytes elevated in 1(3%) patient and LDH increased in 3(6%) patients. Compared to 2 weeks follow-up, one child developed both low fever and headache during the 4 weeks follow-up, but it disappeared quickly. 3(9%) children had runny noses, one of them got mild cough, and 4(12%) children had elevated white blood cells and lymphocytes. Transaminase, uric acid and creatinine were normal, however, LDH and CK respectively increased in 3 (9%) patients and 1 (3%) patient at 2 weeks follow-up compare to 4 (12%) patients and 5 (15%) patients at 4 weeks of follow-up. 2 (6%) patients had slightly elevated CRP at 4 weeks of follow-up. Two weeks follow-up identified normal chest radiographs in 33 (69%) pediatric patients. RT-PCR detection of SARS-CoV-2 were negative in all follow-up patients at 2 and 4 weeks follow-up. All 48 pediatric patients were visited by calling after 1 year of discharge. The outcomes have been favorable including intermittent fever, recurrent respiratory infections, exercise tolerance and psychologic status.

## Discussion

Hunan province is adjacent to Hubei province (of which Wuhan is the capital city), and as of 24:00 on June30, 2020, a total of 1,019 confirmed cases of COVID-19 had been reported there. The government authorities made unprecedented and effective efforts to reduce the risk of transmission. Early diagnosis, early isolation, and early management all contributed to reducing transmission and mortality in Hunan.

Person-to-person transmission and clusters are important features of this epidemic, and close family contact is the main channel of COVID-19 in children. Of the 16 imported cases included in this study, a clear history of exposure in Wuhan and Other cities in Hubei was evident for 15 children; one child was exposed to the virus on a cruise ship. No severe cases have been found in local cases. Of all the 48 cases in this study, 46 children (96%) had been exposed to confirmed adult patients; the other 2 were imported cases, in children who had been living in Wuhan. Compared to adults, children had atypical symptoms or were asymptomatic. In our study, 23% cases were asymptomatic, indicating that the absence of clinical symptoms cannot rule out the diagnosis of infection. It is likely that this explains why so few cases of COVID-19 have been reported in children: asymptomatic pediatric cases are easily missed. Therefore, a detailed investigation of exposure to SARS-CoV-2 is particularly important for children. In our study, there was no significant difference in incidence of COVID-19 between male and female patients. Of the 48 children in our study, 46 (96%) were either asymptomatic, or mild or moderate cases, with only 2 children having severe disease: 1 aged two years and 1 aged four years, both were imported cases. The first critical pediatric patient with COVID-19 reported in China was one year of age (13). Although the incidence of severe and critical illness of COVID-19 in children is low, it appears possible that the younger the age, the higher the incidence of severe and critical disease. This may be related to the fact that at a younger age, organ functions are not fully developed and complications are likely to occur.

Most children with COVID-19 in our study had mainly upper respiratory tract symptoms, such as fever, cough, fatigue, headache, nasal obstruction, and runny nose. Progression to lower respiratory tract infection was present in 46% of children. Gastrointestinal symptoms can be the only clinical manifestation of COVID-19 in children, illustrated by one child in this study, who presented with diarrhea as the only symptom in the course of the disease. Therefore, attention should be paid to children without significant respiratory symptoms. SARS-CoV-2 is rarely reported to involve the nervous system. In this study, a two-year-old girl without any underlying disease developed convulsions in the early stages of COVID-19; these were accompanied by high fever (>39°C) and did not recur after the fever had gone. Although this child developed a severe case with typical ground glass opacity in both lungs, she did not show any neurological abnormalities. We therefore conclude that this was a febrile convulsion and was unrelated to COVID-19.

Lymphocytopenia is common after the onset of COVID-19 in adults but is rare in children. In the initial routine blood tests of this pediatric group, there were only five cases with lymphocytopenia. Thus, for children, especially young children, lymphocytopenia lacks the sensitivity to inform an early diagnosis of COVID-19. On the whole, other laboratory findings in children with COVID-19 in this study showed no significant specificity and sensitivity and could not provide strong evidence for the diagnosis; they could, however, be used as reference indicators to exclude other diseases. CRP level is an indicator for assessing inflammation, and elevated CRP has been found to be present in 60.7% of adult COVID-19 patients, particularly in severe cases (1). In our study, only three children (6%) had elevated CRP, two of which were severe and had elevated procalcitonin. It is likely that patients with severe COVID-19 are more susceptible to secondary bacterial infection than patients with mild disease.

Our study showed some common CT imaging features in patients infected by SARS-CoV-2, including ground glass opacity of bilateral lobular and subsegmental areas of consolidation or mixed shadowing without pleural effusion (white lung appearance). Although the abnormal manifestations of chest CT in some children were typical, they still need to be differentiated from other forms of viral pneumonitis. Therefore, pediatricians should carefully verify findings according to epidemiological history, clinical manifestations, viral nucleic acid detection, and CT images to improve diagnostic accuracy in children.

At present, there is no specific drug treatment for COVID-19. Based on “Diagnosis and prevention of 2019 novel coronavirus infection in children (trial version 1),” the use of LPV/RTV or ribavirin and interferon are recommended. Due to the differences in treatment regimens, dosages, and combinations of antiviral drugs used in the different hospitals included in this study, it was difficult to determine the efficacy of any antiviral drug. The main treatment is symptomatic and supportive care. If a child has a combined secondary bacterial infection, appropriate antibiotics may be used. It is highly recommended that appropriate antimicrobial agents should be selected after a full microbiological investigation, and timely adjustment should be made according to the curative effect and drug sensitivity test results, avoiding blind or inappropriate use of antibiotics. Based on the experience of adult treatment in Wuhan city, routine use of corticosteroids should be avoided and reserved only for severe cases with persistent high fever, CRP ≥ 30 mg/L, serum ferritin ≥1,000 μg/L, or diffuse lesions in both lungs (3). The efficacy of IVIG for COVID-19 pneumonia is limited in adult patients. In this study, after IVIG treatment, all the pulmonary lesions of the two severe cases were resolved. More data is yet required to determine whether the effect of IVIG in children is greater than that in adults.

In this study, due to the limited number of cases in the 12 hospitals and the short study time, we did not collect all pediatric cases in Hunan province. As our understanding of the clinical characteristics and imaging manifestations of COVID-19 in pediatric patients may not yet be comprehensive, a future study with larger sample size from multiple centers is needed.

COVID-19 has been ongoing for only 1 year, it is difficult to identify and investigate the long-term sequelae of SARS-CoV-2 infection. Our follow-up investigation found that the clinical symptoms of pediatric patients basically disappeared within 2 weeks of the follow-up. All patients made complete recoveries with no clinical evidence of residual lung disease and normal follow-up chest radiographs. At the fourth week of follow-up, few patients developed symptoms such as fever, headache, cough, and runny nose during the period, which were not present at 2 weeks follow-up, and was considered to be related to upper respiratory tract reinfection caused by other pathogens. Therefore, our experience suggests that the one year prognosis of children infected with SARS-CoV-2 infection is favorable, it is similar to the follow-up study of coronavirus infection in children in 2003 (14, 15).

In conclusion, the epidemiological characteristics of COVID-19 in 48 children in Hunan province in this study were mainly clustered cases. Most pediatric cases of COVID-19 in Hunan province were asymptomatic, mild, or moderate. Severe cases were rare. Fever and cough were the main symptoms in children with COVID-19. Gastrointestinal symptoms tended to occur in younger children. Epidemiological history, nucleic acid test, and chest imaging were important tools for the diagnosis of COVID-19 in children. All pediatric patients recovered well within one year of follow-up.

## Data Availability Statement

The original contributions generated for the study are included in the article/supplementary material, further inquiries can be directed to the corresponding author/s.

## Ethics Statement

The studies involving human participants were reviewed and approved by Changsha Central Hospital Ethics Committee. Written informed consent to participate in this study was provided by the participants' legal guardian/next of kin. Written informed consent was obtained from the individual(s), and minor(s)' legal guardian/next of kin, for the publication of any potentially identifiable images or data included in this article.

## Author Contributions

LW and M-ZW: conceptualization and writing – original draft preparation. YY, X-YY, X-PJ, H-YH, X-YC, and T-MW: collected the data, formal analysis, and investigation. X-FZ: methodology and writing – review and editing. All authors contributed to the article and approved the submitted version.

## Funding

This work was supported by the COVID-19 special project of Changsha Science and Technology Plan, Grant/Award Number: kq2001011.

## Conflict of Interest

The authors declare that the research was conducted in the absence of any commercial or financial relationships that could be construed as a potential conflict of interest.

## Publisher's Note

All claims expressed in this article are solely those of the authors and do not necessarily represent those of their affiliated organizations, or those of the publisher, the editors and the reviewers. Any product that may be evaluated in this article, or claim that may be made by its manufacturer, is not guaranteed or endorsed by the publisher.
